# A Microarray Study of Middle Cerebral Occlusion Rat Brain with Acupuncture Intervention

**DOI:** 10.1155/2015/496932

**Published:** 2015-03-10

**Authors:** Chao Zhang, Yan Wen, Xiaonong Fan, Sha Yang, Guang Tian, Xueyi Zhou, Yaqiong Chen, Zhihong Meng

**Affiliations:** ^1^Department of Acupuncture and Moxibustion, First Teaching Hospital of Tianjin University of Traditional Chinese Medicine, Tianjin 300193, China; ^2^Acupuncture and Moxibustion Research Institute of Tianjin University of Traditional Chinese Medicine, Tianjin, China; ^3^Key Laboratory of Cerebropathy Acupuncture Therapy of State Administration of Traditional Chinese Medicine, Tianjin 300193, China; ^4^Tianjin Key Laboratory of Acupuncture & Moxibustion Science, Tianjin 300193, China; ^5^Three-Level Laboratory of Acupuncture Dose-Effect Relationship, State Administration of Traditional Chinese Medicine, Tianjin 300193, China; ^6^Post-Graduate School, Tianjin University of Traditional Chinese Medicine, Tianjin 300193, China

## Abstract

Microarray analysis was used to investigate the changes of gene expression of ischemic stroke and acupuncture intervention in middle cerebral artery occlusion (MCAo) rat brain. Results showed that acupuncture intervention had a remarkable improvement in neural deficit score, cerebral blood flow, and cerebral infarction volume of MCAo rats. Microarray analysis showed that a total of 627 different expression genes were regulated in ischemic stroke. 417 genes were upregulated and 210 genes were downregulated. A total of 361 different expression genes were regulated after acupuncture intervention. Three genes were upregulated and 358 genes were downregulated. The expression of novel genes after acupuncture intervention, including *Tph1* and *Olr883*, was further analyzed by Real-Time Quantitative Polymerase Chain Reaction (RT-PCR). Upregulation of *Tph1* and downregulation of *Olr883* indicated that the therapeutic effect of acupuncture for ischemic stroke may be closely related to the suppression of poststroke depression and regulation of olfactory transduction. In conclusion, the present study may enrich our understanding of the multiple pathological process of ischemic brain injury and indicate possible mechanisms of acupuncture on ischemic stroke.

## 1. Introduction

Stroke is still a worldwide disease today which causes millions of deaths and more numbers of physical disabilities every year [1, 2]. It is also the leading cause of death and long-term disability in China, where the annual stroke mortality rate is approximately 1.6 million. There are over 7 million stroke survivors in the country with 70% of them being ischemic [3, 4]. The complexity of pathological changes involved in the process of ischemic stroke, worsened by the narrow treatment window (3–6 hs) of rt-PA therapy and the risk of hemorrhage of antiplatelet therapy and anticoagulation therapy [5], has led to the unoptimistic situation of the treatment of ischemic stroke. Sequela syndromes are commonly seen in ischemic stroke patients; thus physical training (PT) and other complementary therapy are increasingly used in ischemic stroke patients.

Acupuncture is a traditional Chinese medical therapy which has been proved effective in the treatment of ischemic stroke [6–9]. Researches have found that acupuncture can effectively improve the motor function of ischemic stroke patients [10], possibly due to the improvement of cerebral circulation [11–13], but the underlying biological mechanisms of acupuncture effects are still poorly understood and need to be further explored.

Microarray research has been a hot topic since the last decade. Gene-chips provide the means to measure simultaneously where and when thousands of genes are expressed [14]. This technology allows detection and quantification of the differential expression of thousands of genes simultaneously in a single experiment. This technology has helped illuminate mechanisms of disease and identify disease subphenotypes, predict disease progression, assign function to previously unannotated genes, and group genes into functional pathways [15]. Thus, many researches have tried to explore the secret of stroke by using gene-chip technology [16–21] and a lot of biological processes have been found to be related to the pathological injury of ischemic stroke such as inflammation [22], apoptosis [20], and excitotoxicity [23]. So we assume that the effect of acupuncture on ischemic stroke may be acquired from the regulation of these biological processes. In the present study, gene-chip technology was used to analyze the gene expression in ischemic stroke and acupuncture intervention to find out how acupuncture regulates the gene expression of ischemia brain tissues in the model of MCAo rats and thus to reveal possible biological mechanisms of acupuncture on ischemic stroke.

## 2. Experimental Procedures

### 2.1. Experimental Groups and MCAo Model

**Figure 1:**
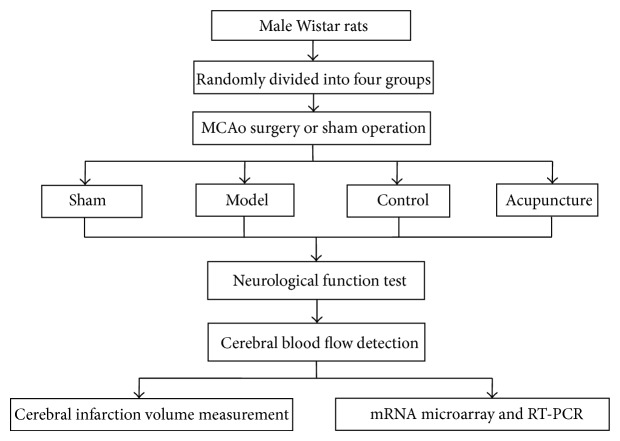
Schematic diagram for methodologies: a total of 72 adult male Wistar rats were randomly divided into the sham, model, control, and acupuncture groups. Rats in the model group underwent the measurements of neurological function, cerebral blood flow (CBF), and cerebral infarction volume immediately after they regained consciousness from anesthesia while rats in other groups received the measurements 60 hours later. Six rats were randomly selected from each group for mRNA microarray and RT-PCR test and 12 others for cerebral infarction volume measurement.

Male Wistar rats weighing 250–300 g, aged 3 months, were selected from the Laboratory Animal Center of People's Liberation Army Academy of Military Medical Sciences, Beijing, China [License no. SCXK-(Army) 2012-0004]. Animals were acclimated to the animal quarters at least for 3 days before the experiment and were provided with a standard laboratory diet and water ad libitum. Animal treatments were performed in a manner to minimize pain or discomfort in accordance with the Guidance Suggestions for the Care and Use of Laboratory Animals, formulated by the Ministry of Science and Technology of China. Rats were randomly divided into 4 groups including model, sham, control, and acupuncture. The minimum number of animals (*n* = 18) was used for each group. Rats in the model, control, and acupuncture groups were subjected to MCAo surgery. The difference between the model and control groups was that rats in the model group underwent neurological function test, cerebral blood flow detection, and cerebral infarction volume measurement immediately after they recovered from anesthesia while rats in the control group received all these measurement 60 hours later together with the acupuncture group. The experimental protocols were shown in Figure 1. A modified thread ligation method invented by Longa et al. [24] was applied to duplicate the model of middle cerebral artery occlusion. Briefly, rats were fasted for 12 hours with free access to water and anesthetized by intraperitoneal injection of 10% chloral hydrate (350 mg/kg). Rats were then fixed in the dorsal position on the surgery board, neck skin and muscle were incised, and the common carotid artery, the external carotid artery, and the internal carotid artery on the left were isolated. The external carotid artery and the proximal end (near the heart) of the common carotid were ligatured with 0-suture line. A small hole was pierced with a 1 mL syringe needle at the proximal end of the common carotid. A 0.28 mm nylon thread was inserted from the hole into the internal carotid until resistance was met, with an intracranial depth of 18–20 mm. Blood flow in the left middle cerebral artery was blocked by the nylon thread. The nylon thread was then ligatured with the common carotid artery and muscle and skin were sutured. The nylon thread was not applied in rats of sham group. This intraluminal suture model of MCAo produces reliable and permanent focal cerebral ischemia.

### 2.2. Acupuncture Intervention Methods

To rats in the acupuncture group, Neiguan (PC 6) acupoint on the right forelimb was needled with twisting-rotating method in the frequency of 3 times per second for 60 seconds. According to the Acupoint Location of Commonly Used Experimental Animals in the Experimental Acupuncture Science [25], Neiguan (PC 6) acupoint was located at the forelimb, between the ulna and the radius, about 3 mm from the wrist, perpendicularly needled 2 mm in depth. Sterile disposable stainless steel needles (length: 40 mm, diameter: 0.30 mm; Hwato, Suzhou Medical Supplies Factory Co., Ltd., China) were used in this study. All the acupuncture manipulation was operated by a skilled acupuncturist. Rats were needled for the first time right after they recovered from MCAo surgery and received 5 more times of acupuncture treatment in the subsequent 60 hours, 12 hours once.

To the sham and control groups, rats did not receive any acupuncture intervention but were also grabbed six times with the acupuncture group in the experimental period. To the model group, rats did not receive any acupuncture intervention. They received neurobehavioral test and cerebral blood flow (CBF) detection as soon as they recovered from anesthesia and then their brains were immediately collected for cerebral infarction volume measurement and microarray test.

### 2.3. Neurological Function Test

Neurological function test was performed according to Zausinger's six-point methods [26] once rats recovered from anesthesia. The standards used to obtain Zausinger's six-point score were as follows: score 0, the rat could not spontaneously walk; score 1, the rat rotated towards the side opposite to the lesion with free walking; score 2, the rat rotated towards the side opposite to the lesion when its tail was seized; score 3, the resistance to the lateral pressure was decreased in the side opposite to the lesion; score 4, the rat could not unbend front paws or entire forelimb on the side opposite to the lesion; score 5, the rat had no neurological function defect. Only rats with a score of 1–3 were considered to be successful cerebral ischemia models and rats with a score of 0, 4, or 5 were not used for further experimentation. At 60 hours after modeling, all rats were assessed for another time except for the model group.

### 2.4. CBF Detection

CBF was measured by laser Doppler flowmetry (DRT4, Moor Instrument, Wilmington, DE, USA). Rats were fixed on a self-made stereotaxic instrument after anesthesia. A midline incision was made on the scalp to expose the anterior fontanel. A small bone window 1 mm posterior to the anterior fontanel and 3 mm left to the midline was produced with a dental drill. The measurement probe was placed on the left cerebral hemisphere to monitor moving red blood cells. CBF was continuously measured for 1 minute for each rat. Rats in the model group were immediately measured after they recovered from anesthesia. Rats in the sham and control groups were measured 60 hours later together with the acupuncture groups.

### 2.5. Brain Collection and Cerebral Infarction Volume Measurement

After neurological function test and CBF detection, six rats were randomly selected from each group for microarray test and others were conducted for cerebral infarction volume measurement. All rats were euthanized with chloral hydrate and their brains were collected quickly. As to the rats for microarray test, their left hemispheres were separated and kept at a temperature of −80°C for mRNA extraction later. To other rats, their brains were placed at a temperature of −20°C for 30 minutes and each then was cut into 5 coronal sections each 3 mm thick and stained with a 2% solution of 2,3,5-triphenyltetrazolium chloride (TTC) in phosphate buffered saline (PBS) at a temperature of 37°C for 20 min, followed by 4% paraformaldehyde buffer for fixation. The stained sections were photographed by Olympus fe-240 digital camera (Pooher Photoelectric Technology Co., Ltd., Shanghai, China) and the digital images were analyzed by a computer-assisted image system with Image-Pro Plus 6.0 (Rockville, MD, USA). The infarction volume was presented as a percentage of the total ipsilateral hemispheric volume which can be calculated by following equation: [(the volume of the intact contralateral hemisphere) – (the volume of the intact ipsilateral hemisphere – the volume of infarcted tissue in the ipsilateral hemisphere)]/(the volume of the intact contralateral hemisphere) × 100%. This rectified measurement equation corrects for edema in the total infarct volume.

### 2.6. RNA Extraction

Total RNA was extracted from ipsilateral hemispheres of MCAo rats and the corresponding hemispheres of sham rats with TRIZOL Reagent (GibcoBRL, Rockville, MD) according to manufacturers' protocol and its quality was measured by NANODROP 2000 spectrophotometer (Thermo Scientific, USA) and 2100 Bioanalyzer (Agilent Technologies, Inc., USA). The total RNA was then purified with Qiagen RNeasy Mini Kit (Qiagen Inc., USA) and quantified by spectrophotometer and stored at −80°C for microarray analysis and Real-Time Quantitative Polymerase Chain Reaction (RT-PCR) studies.

### 2.7. Microarray Analysis

An equal amount of total RNA (100 ng) was used for the synthesis of cDNA and then cDNA was cleaned and used for cy3CTP, cy5CTP-labeled cRNA synthesis with T7 RNA polymerase. The labeled and fragmented cRNA was then hybridized to Agilent G4853A Gene-Chip (Agilent Technologies, Inc., USA) which contains 30,003 functional genes for 17 hours with required antisense controls. The microarray chips were then washed and scanned using Agilent Scan Control software (Agilent Technologies, Inc., USA) and data was analyzed by Agilent GeneSpring software GX10.05 (Agilent Technologies, Inc., USA). Differentially expressed genes were selected based on *P* value <0.05 and log⁡_2_⁡ (Fold Change) ≥ 1 or log⁡_2_⁡ (Fold Change) ≤ −1.

### 2.8. KEGG Pathway Analyses for mRNAs

The Kyoto Encyclopedia of Genes and Genomes (KEGG) is a knowledge base for systematic analysis of gene functions, linking genomic information with higher order function information. KEGG pathway database is supplemented by a set of ortholog group tables for the information about conserved subpathways (pathway motifs), which are often encoded by positionally coupled genes on the chromosome and are especially useful in predicting gene functions [27]. Differently expressed genes were used for KEGG pathway analysis between groups (model versus sham, control versus acupuncture). Hypergeometric Distribution method was applied to calculate the *P* value of each pathway and then rectified by false discovery rate (FDR) method. Possibility values of <0.05 were considered statistically significant.

### 2.9. Real-Time Quantitative Polymerase Chain Reaction Validation

Real-Time Quantitative Polymerase Chain Reaction (RT-PCR) was carried out for mRNA validation according to manufacturer's protocols using mRNA specific primers from Applied Biosystems (USA). Two genes, that is,* Tph1* and* Olr883,* were chosen from all the differentially expressed genes. The complementary DNAs were synthesized by Shanghai Science & Technical Co. (Shanghai, China). All samples were normalized by* Rattus norvegicus* actin. The information about primers used for RT-PCR was shown in Table 1. Each sample was tested in triplicate. Relative gene expression was measured by 2^−ΔΔCT^ method [28].

### 2.10. Statistical Analysis

All data in our study were analyzed by SPSS 16.0 software and presented as mean ± SD except for microarray analysis. One-way analysis of variance (ANOVA) followed by the least significant difference (LSD) was used for analyzing the data. The standard statistical function of Hypergeometric Distribution, *t*-test, and FDR was performed for determined genes differently expressed in microarray analysis and KEGG pathways. Possibility values of <0.05 were considered statistically significant.

## 3. Results

### 3.1. Acupuncture Improved Neurological Function of MCAo Rats

**Figure 2:**
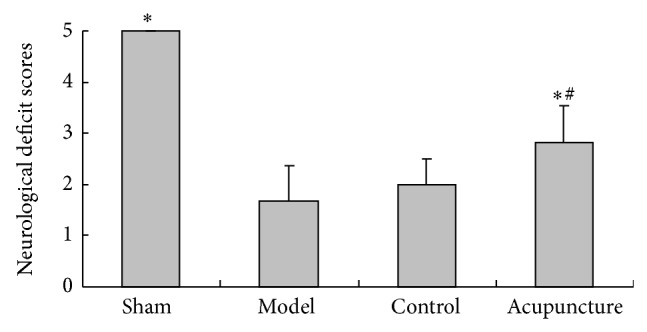
Effects of acupuncture on neurological deficit scores in MCAo rats: values are mean ± SD (*n* = 18) and ^*^ *P* < 0.05 compared with the model group, ^#^ *P* < 0.05 compared with the control group.

Compared with the sham group, neurological deficit scores significantly decreased in the model group (*P* < 0.05) but improved after 60 hours in the acupuncture group (*P* < 0.05). No significant difference was seen between the model and control groups (*P* > 0.05) while the acupuncture group had an apparent improvement compared with the control group (*P* < 0.05) (see Figure 2).

### 3.2. Acupuncture Increased Cerebral Blood Flow (CBF) of MCAo Rats

**Figure 3:**
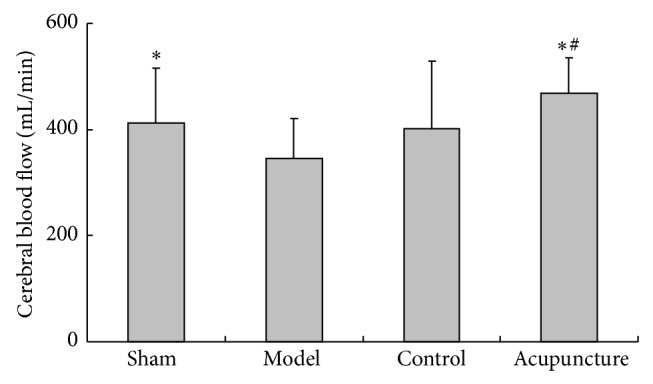
Effects of acupuncture on CBF in MCAo rats: values are mean ± SD (*n* = 18) and ^*^ *P* < 0.05 compared with the model group, ^#^ *P* < 0.05 compared with the control group.

CBF was significantly reduced in the model control group compared with the sham group (*P* < 0.05) and increased after 60 hours in the acupuncture group (*P* < 0.05). No significant difference was seen between the model and control groups (*P* > 0.05) while the acupuncture group had an apparent increase compared with the control group (*P* < 0.05) (see Figure 3).

### 3.3. Acupuncture Reduced Cerebral Infarction Volume of MCAo Rats

**Figure 4:**
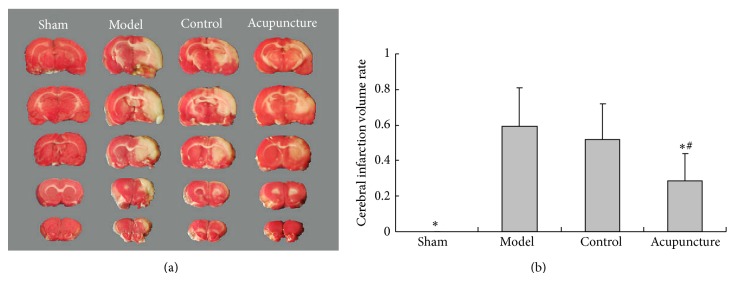
Effects of acupuncture on infarction volume. (a) Representative coronal sections of rat brains stained by TTC from four groups. The white area is infarct region and red is normal tissue. (b) Quantitative analysis of infarction volume. Values are mean ± SD (*n* = 12) and ^*^ *P* < 0.05 compared with the model group, ^#^ *P* < 0.05 compared with the control group.

Compared with the sham group (zero), clear cerebral infarction volume was seen in the model group (*P* < 0.05) and reduced in the acupuncture group (*P* < 0.05). No significant difference was seen between the model and control groups (*P* > 0.05) (see Figure 4).

### 3.4. Changes in mRNA Expression and Pathways Involved in the Process of Ischemic Stroke

A total of 627 different expression genes were regulated during the process of ischemic stroke. 417 genes were upregulated and 210 genes were downregulated.

Among the upregulated genes,* Hspa1b*,* Ccl2*,* Cxcl2*,* Ccl3*,* Serpina3n*,* Timp1*,* H19*,* Hspb1*,* Ccl20*, and* Cxcl1 *were found to demonstrate significant upregulation in pathological phase of ischemic stroke (see Table 2). 58 pathways were examined for upregulated mRNAs in this study including the regulation of* Staphylococcus aureus* infection, phagosome, cell adhesion molecules, cytokine-cytokine receptor interaction, hematopoietic cell lineage, Toll-like receptor signaling pathway, natural killer cell mediated cytotoxicity MAPK signaling pathway, p53 signaling pathway, VEGF signaling pathway, and TGF-beta signaling pathway (see Table 3).

Among the downregulated genes,* Gpr88*,* Rgs9*,* Enthd1*,* Olr59*,* P2ry12*,* Degs2*,* Pde10a*,* Neu2*,* Adora2a*, and* Slc22a6* were found to demonstrate significant downregulation in pathological phase of ischemic stroke (see Table 4). 19 pathways of downregulated mRNAs were examined including the regulation of calcium signaling pathway, gastric acid secretion, salivary secretion, axon guidance, hypertrophic cardiomyopathy, neuroactive ligand-receptor interaction, long-term depression, axon guidance, pancreatic secretion, and sphingolipid metabolism (see Table 5).

### 3.5. Changes in mRNA Expression and Pathways Involved in Acupuncture Intervention

A total of 361 different expression genes were regulated during the process of acupuncture intervention. Three genes were upregulated and 358 genes were downregulated.

The three upregulated genes were* Tph1*,* Loc684158, *and* Ccdc19 *(see Table 6). No pathway was found among the 3 upregulated mRNAs, so pathway annotation was adopted and two pathways were involved, that is, tryptophan metabolism and metabolism (see Table 7).

Among the downregulated genes,* Gpr88*,* Rgs9*,* Enthd1*,* Olr59*,* P2ry12*,* Degs2*,* Pde10a*,* Neu2*,* Adora2a*, and* Slc22a6* were found to demonstrate significant downregulation in pathological phase of ischemic stroke (see Table 8). The pathway of acupuncture intervention for downregulated mRNAs examined in this study is olfactory transduction (see Table 9).

### 3.6. Verification of Gene Expression By RT-PCR

**Figure 5:**
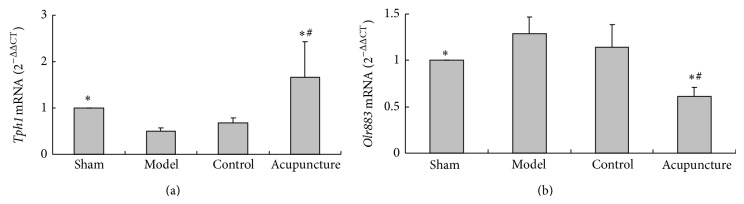
Analysis of mRNA expression of* Tph1* and* Olr883* by RT-PCR. (a) mRNA expression of* Tph1* in the sham, model, control, and acupuncture groups. (b) mRNA expression of* Olr883* in the sham, model, control, and acupuncture groups. Values are mean ± SD (*n* = 6) and ^*^ *P* < 0.05 compared with the model group, ^#^ *P* < 0.05 compared with the control group.

RT-PCR results of* Tph1* and* Olr883* showed that directional changes of mRNA levels were in agreement with microarray analysis results. mRNA expression of* Tph1* was reduced in the model group and increased in the acupuncture group (*P* < 0.05). No difference was seen between the control and model groups (*P* > 0.05) (see Figure 5(a)). mRNA expression of* Olr883* was increased in the model group and reduced in the acupuncture group (*P* < 0.05). No difference was seen between the control and model groups (*P* > 0.05) (see Figure 5(b)).

## 4. Discussion

To better understand the mechanisms of the therapeutic effect of acupuncture on ischemic stroke, we used microarray analysis in rat brain to examine the genomic response to ischemic stroke and acupuncture intervention. To the best of our knowledge, this is the first report using an mRNA microarray to survey global differential gene expression after manual acupuncture treatment in ischemic stroke animal models.

The microarray analysis performed during the period of ischemic stroke confirmed the bidirectional expressions of many genes. The gene expression of ischaemic stroke is characterised by differential expressions of a great number of genes involved in inflammation, apoptosis, protein turnover, transcription, signal transduction, ion-channel regulation, and metabolism which suggests that ischemic stroke is a multiple pathological process and it can trigger a multifaceted cascade of physiologic and biochemical events. These events are mediated in part by alterations of molecular transcriptional and translational activities. Our microarray findings are in accordance with previous studies of individual genes and pathways proposed to be associated with ischaemic brain injury [29, 30]. Most of the differential genes were upregulated including heat shock protein (Hsp) genes and chemokine ligand genes. The upregulation of* Hsp 70 *genes such as* Hspa1b* and* Hspa1 *after ischemic stroke in the present study is consistent with other studies [29, 31]. As we all know, apoptosis-related genes are induced after focal ischemia, and they may contribute to cell death in the core and the selective cell death adjacent to an infarct [32], whereas heat shock proteins have been shown to play an important protective role in vivo as they have the ability to protect cultured cells from apoptosis and thus to protect brain against ischaemia [33]. Similar to heat shock proteins, an increase in mRNA expression of chemokines (*Ccl2*,* Ccl3*,* Cxcl2*, and* Ccl20*) was observed in the present study and this result is also consistent with other studies [31, 34]. Members of the CC and CXC chemokines are induced in the rat brain after focal cerebral ischemia and they are believed to contribute to secondary neuronal damage [29] through modulating crucial processes such as inflammatory cell recruitment and activation, neuronal survival, and neoangiogenesis. On the other hand, CXC (*Cxcl2*,* Cxcl3*) chemokines could also modulate stem cell homing, thus favouring tissue repair [35]. Therefore chemokine receptors were potential targets for therapeutic intervention in stroke [36]. Upregulated chemokines in the ischemic brain may reduce inflammatory cell migration to the brain in early stroke. Inhibition of inflammatory cell accumulation in the brain at the early stage of stroke may lead to amelioration of ischemic neurodegeneration [37, 38].

Compared with upregulated genes of ischemic stroke, information on downregulated genes seems relatively limited. Among the genes that were downregulated, G-protein genes were found to be closely related to ischemic injury. The signal transduction system mediated by G-protein is the most common way of signaling in cells. According to our microarray results, G-protein genes such as* Gpr88 *and* Rgs9* were downregulated.* Gpr88 *may play a role in the fundamental functions of striatum such as the control of motor behavior [39]. A recent study reported that, in* Gpr88* knockout mice, basal extracellular dopamine levels in the striatum were lower, while amphetamine-induced dopamine release was normal, and these mice also displayed increased apomorphine-induced stereotypy and amphetamine-stimulated locomotor activity [40]. These results suggest that* Gpr88* plays a role in the regulation of dopamine signaling in the striatum and it may be a new target for treatments for psychiatric disorders after ischemic stroke. The* Rgs9* gene gives rise to two splice forms* Rgs9-1 *and* Rgs9-2*.* Rgs9-1* is expressed exclusively in retina, while* Rgs9-2* is highly enriched in striatal regions of the brain.* Rgs9 *plays a role in striatal dopamine-mediated behavior [41] and has been shown to be a critical negative regulator of opiate action in vivo [42].* Rgs9* knockout rats exhibit normal locomotor activity, anxiety-like behavior, cued and contextual fear conditioning, startle threshold, and prepulse inhibition which suggest that* Rgs9-2* is a potential therapeutic target for disorders involving motor or cognitive dysfunction [43].

Among all the different expression genes in the process of ischemic stroke, some may aggravate the obstruction of blood flow, death of ischemic neurons, and dysfunction of subsequent neurological behavior that had led to the early and ultimate pathological injury of ischemic stroke and some may play an important protective role as the spontaneous anti-injury response in the pathological process of ischemic stroke. The microarray results also showed that ischemic stroke had a widespread influence on the whole body rather than a specific change in the brain; therefore therapeutic strategy aiming at the remedy of single pathological change might not obtain optimistic result.

According to previous researches, spontaneous recovery plays an important role in the pathological process of ischemic stroke [44]. In the present study, we observed the spontaneous recovery in 60 hours after modeling in MCAo rats; however, results did not show any sound outcomes. On the other hand, acupuncture intervention could effectively improve the neurological function and CBF and reduce the infarction volume of MCAo rats (see Figures 2, 3, and 4). Different patterns of gene expression occurred in the control and acupuncture groups. Our microarray analysis showed that most gene expression levels were downregulated following acupuncture treatment which suggests that the therapeutic effect of acupuncture on ischemic stroke may mainly depend on strategies to suppress the pathological progress of ischemic stroke instead of facilitating the repair of ischemic stroke injury. KEGG pathway analysis showed that the only pathway of the downregulated genes was olfactory transduction. Olfactory ensheathing cells (OECs) express adhesion molecules on their plasma membranes to help neural cells attach, and they also secrete a variety of trophic factors to facilitate neural regeneration and axon outgrowth. Intracerebral transplantation of OECs/olfactory nerve fibroblasts (ONFs), which secrete trophic factors including stromal cell-derived factor-1a (SDF-1a), effectively leads to the recovery of the damaged cerebral tissue in murine models of stroke, thereby promoting the reversal of neurological deficit [45]. Transplantation of OECs has been shown to protect the white matter from ischemic injury [46], leading to improving neurological function in rats. Although there is relatively limited research of olfactory transduction in ischemia stroke, our result suggests that olfactory transduction might play an important role in ischemic stroke recovery and the therapeutic effect of acupuncture on ischemic stroke is closely related to the regulation of olfactory transduction.

Three genes were found upregulated after acupuncture intervention including* Tph1*,* Loc684158,* and* Ccdc19*. Pathway annotation showed tryptophan metabolism was concerned.* Tph1* is a subtype of tryptophan hydroxylase (TPH) and TPH plays an important role in the biosynthesis of 5-hydroxytryptamine (5-HT). Cerebral ischemia disturbs the normal metabolism of amine and decreases the bioavailability of amine in brain tissues which causes the hyposecretion of 5-hydroxytryptamine (5-HT), norepinephrine (NE), dopamine, and other neurotransmitters [47]. This kind of amine metabolism closely related to the mechanism of poststroke depression. At least 1/3 stroke survivors have varying degrees of emotional symptoms [48, 49]. Depression is the most common symptom which increases the mortality, recurrence rate, and mobility of stroke patients [50–53]. TPH starts the synthesis process by catalyzing L-tryptophan into 5-HT and also acts as the rate limiting enzyme in the whole process. Therefore the activity of TPH directly influences the level and function of 5-HT. Our result of microarray analysis and RT-PCR result showed that the expression of* Tph1* was increased after acupuncture treatment which indicated that acupuncture had a potential effect on poststroke depression by promoting the level of 5-HT in brain.

In conclusion, the present study discovered several characteristics of cerebral ischemia-induced gene expression and they may enrich our understanding of the multiple pathological process of ischemic brain injury. Meanwhile we also discovered therapeutic candidates stimulated or inhibited by acupuncture in ischemic stroke, and two genes were especially investigated. Upregulation of* Tph1* and downregulation of* Olr883* after acupuncture treatment indicate that the therapeutic effect of acupuncture for ischemic stroke may be closely related to the suppression of poststroke depression and regulation of olfactory transduction. These results may be useful in pursuing further studies on the possible mechanisms of acupuncture on ischemic stroke. Considering the difference between rats and human beings [54], subsequent clinical researches are also needed to further approve the findings of the current study.

## Supplementary Material

Supplementary figure 1 shows the different expression genes of the model group versus the sham group.
Supplementary figure 2 presents the enriched pathways of the upregulated genes of the model group versus the sham group.
Supplementary figure 3 presents the enriched pathways of the downregulated genes of the model group versus the sham group.
Supplementary figure 4 shows the different expression genes of the acupuncture group versus the control group.
Supplementary figure 5 presents the enriched pathways of the downregulated genes of the acupuncture group versus the control group.
Supplementary figure 6 gives the melting curves of Tph1 and Olr883 in RT-PCR experiment: (a) Melting curve of Tph1. (b) Melting curve of Olr883.

## Figures and Tables

**Table 1:** Primer names, sequences, and PCR conditions used for RT-PCR analysis.

Gene	NCBI reference sequence	Primer sequence	Tm (°C)	Amplicon size (bp)
*Tph1 *	NM_001100634.2	5′GGCTTTGAGGTCCTCTTTCCA3′	56	112
		5′CCCCCTTTCTGAGGAATGGTC3′		

*Olr883 *	NM_001001358.1	5′GCAGGCCACTGCACTATTTG3′	56	123
		5′ACTGCAGATTTAGGCCGAGG3′		

*Actin *	NM_031144.3	5′CAGCCTTCCTTCCTGGGTATG3′	55	247
		5′TAGAGCCACCAATCCACACAG3′		

**Table 2:** The top 10 upregulated genes of the model group versus the sham group.

Gene symbol	Gene description	log_2_⁡(FC)	*P* value	*P*.adjust
*Hspa1b *	Heat shock 70 kD protein 1B (mapped)	6.91524	3.64*E* − 07	3.96*E* − 04
*Ccl2 *	Chemokine (C-C motif) ligand 2	6.85826	1.72*E* − 06	1.16*E* − 03
*Cxcl2 *	Chemokine (C-X-C motif) ligand 2	6.37562	7.87*E* − 05	7.37*E* − 03
*Ccl3 *	Chemokine (C-C motif) ligand 3	5.67107	7.19*E* − 06	2.43*E* − 03
*Serpina3n *	Serine (or cysteine) peptidase inhibitor, clade A, member 3N	5.61058	2.11*E* − 05	4.16*E* − 03
*Timp1 *	TIMP metallopeptidase inhibitor 1	5.60194	7.64*E* − 05	7.34*E* − 03
*H19 *	H19, imprinted maternally expressed transcript (nonprotein coding)	5.43809	4.25*E* − 06	1.80*E* − 03
*Hspb1 *	Heat shock protein 1	5.41504	1.15*E* − 08	5.62*E* − 05
*Ccl20 *	Chemokine (C-C motif) ligand 20	5.20095	3.47*E* − 04	1.51*E* − 02
*Cxcl1 *	Chemokine (C-X-C motif) ligand 1 (melanoma growth stimulating activity, alpha)	5.17590	4.94*E* − 06	1.91*E* − 03

**Table 3:** The enriched pathways of the up-regulated genes of the model group versus the sham group.

KEGG ID	Description	*P* value	Gene name
5144	Malaria	6.08*E* − 14	*Selp; Il6; Ccl12; Il1b; Cd36; Tnf; Tlr2; RGD1565355; Tgfb1; Itgb2; Thbs4; Icam1; Cd40; Sele; Myd88; Sdc1 *
5140	Leishmaniasis	1.15574*E* − 13	*Ncf4; Il1b; Jun; Fcgr3a; Tnf; Tlr2; Ptpn6; Fcgr1a; Fos; Ptgs2; Tgfb1; Itgam; Itgb2; RT1-DMa; Nfkbia; Myd88; LOC498276 *
5150	*Staphylococcusaureus* infection	1.70375*E* − 12	*Selp; C1qc; Fcgr3a; C1s; C3ar1; Fcgr1a; Itgam; Fcgr2b; Itgb2; RT1-DMa; C5ar1; Icam1; LOC498276; C1qb *
5323	Rheumatoid arthritis	5.37292*E* − 11	*Il6; Ccl12; Il1b; Jun; Tnf; Tlr2; Csf1; Cd86; Fos; Ccl20; Tgfb1; Itgb2; RT1-DMa; Icam1; Ccl3; Il11 *
4380	Osteoclast differentiation	5.73512*E* − 11	*Ncf4; Il1b; Jun; Fcgr3a; Tyrobp; Tnf; Lcp2; Spi1; Csf1; Fcgr1a; Fos; Nfkb2; Tgfb1; Fcgr2b; Fosl1; Nfkbia; Socs3; Tnfrsf1a; LOC498276 *
4145	Phagosome	1.7585*E* − 10	*Msr1; RT1-EC2; Ncf4; Fcgr3a; Cd36; RT1-A2; RT1-A1; Tap1; Tlr2; RGD1565355; Fcgr1a; Tubb6; Itga5; Cd14; Itgam; Fcgr2b; Itgb2; RT1-CE5; RT1-DMa; Thbs4; Olr1; RT1-CE2; LOC498276 *
4514	Cell adhesion molecules (CAMs)	2.37222*E* − 10	*Ptprc; RT1-EC2; Selp; RT1-A2; RT1-A1; Cldn14; Cldn23; Cd86; Esam; Itgam; PVR; Itgb2; RT1-CE5; RT1-DMa; Cd274; Icam1; Cd40; Sele; RT1-CE2; Sdc1; Glycam1 *
4060	Cytokine-cytokine receptor interaction	1.65905*E* − 09	*Il6; Ccl12; Il1b; Cxcl16; Tnf; Ccl4; Clcf1; Csf1; Cxcl2; Il1r2; Cxcl10; Tnfrsf12a; Tnfrsf1b; Ccl20; Il4ra; Tgfb1; Ltbr; Il13ra1; Cd40; Tnfrsf1a; Ccl3; Il11; Csf2rb *
4640	Hematopoietic cell lineage	2.81713*E* − 09	*Il6; Il1b; Cd36; Tnf; Csf1; RGD1565355; Fcgr1a; Itga5; Il1r2; Cd14; Il4ra; Itgam; Cd44; Il11 *
4620	Toll-like receptor signaling pathway	3.71149*E* − 09	*Il6; Il1b; Jun; Tnf; Tlr2; Cd86; Cxcl10; Cd14; Fos; Lbp; Cd40; Nfkbia; Ccl3; Myd88; Spp1 *
4610	Complement and coagulation cascades	3.91282*E* − 09	*Serpine1; C1qc; Fga; Kng1; Plaur; C1s; C3ar1; Serping1; A2m; C6; C5ar1; F10; C1qb *
5142	Chagas disease (American trypanosomiasis)	1.28382*E* − 08	*Serpine1; C1qc; Il6; Ccl12; Il1b; Jun; Tnf; Tlr2; Fos; Tgfb1; Nfkbia; Tnfrsf1a; Ccl3; Myd88; C1qb *
4062	Chemokine signaling pathway	7.78649*E* − 08	*Ccl2; Hck; Ccl6; Ccl7; Ccl12; Shc1; Cxcl16; Cxcl1; Ccl4; Cxcl2; Adcy4; Cxcl10; Fgr; Rac2; Ccl20; Stat3; Nfkbia; Ccl3; Jak3 *
5332	Graft-versus-host disease	5.76242*E* − 07	*RT1-EC2; Il6; Il1b; RT1-A2; RT1-A1; Tnf; Cd86; RT1-CE5; RT1-DMa; RT1-CE2 *
4612	Antigen processing and presentation	1.07678*E* − 06	*RT1-EC2; RT1-A2; RT1-A1; Tap1; Tnf; Ifi30; Hspa2; Lgmn; Hspa1b; RT1-CE5; RT1-DMa; RT1-CE2 *
4010	MAPK signaling pathway	1.18683*E* − 06	*Gadd45g; Myc; Il1b; Hspb1; Jun; Flna; Tnf; Flnc; Gadd45b; Fgf2; Hspa2; Il1r2; Cd14; Rac2; Fos; Map3k6; Nfkb2; Tgfb1; Gadd45a; Hspa1b; Tnfrsf1a; Dusp5 *
4621	NOD-like receptor signaling pathway	1.44958*E* − 06	*Il6; Ccl12; Il1b; Cxcl1; Tnf; Birc3; Cxcl2; Pycard; Nfkbia *
5146	Amoebiasis	2.86186*E* − 06	*Il6; Il1b; Hspb1; Tnf; Tlr2; Col4a1; Il1r2; Cd14; Col4a2; Tgfb1; Itgam; Itgb2 *
4512	ECM-receptor interaction	5.01276*E* − 06	*Cd36; Tnc; RGD1565355; Col4a1; Itga5; Col4a2; Thbs4; Cd44; Sdc1; Spp1 *
5330	Allograft rejection	6.43761*E* − 06	*RT1-EC2; RT1-A2; RT1-A1; Tnf; Cd86; RT1-CE5; RT1-DMa; Cd40; RT1-CE2 *
4920	Adipocytokine signaling pathway	1.72078*E* − 05	*Cd36; Tnf; RGD1565355; Tnfrsf1b; Prkab2; Stat3; Nfkbia; Socs3; Tnfrsf1a *
4940	Type I diabetes mellitus	1.72078*E* − 05	*RT1-EC2; Il1b; RT1-A2; RT1-A1; Tnf; Cd86; RT1-CE5; RT1-DMa; RT1-CE2 *
5416	Viral myocarditis	1.7893*E* − 05	*RT1-EC2; RT1-A2; RT1-A1; Cd86; Rac2; Itgb2; RT1-CE5; RT1-DMa; Icam1; Cd40; RT1-CE2 *
4623	Cytosolic DNA-sensing pathway	4.3625*E* − 05	*Il6; Il1b; Ripk3; Ccl4; Cxcl10; Pycard; Nfkbia *
5143	African trypanosomiasis	4.73199*E* − 05	*Il6; Il1b; Tnf; Icam1; Sele; Myd88 *
5145	Toxoplasmosis	5.71133*E* − 05	*Tnf; Tlr2; Birc3; Hspa2; Tgfb1; Stat3; Hspa1b; RT1-DMa; Cd40; Nfkbia; Tnfrsf1a; Myd88 *
5322	Systemic lupus erythematosus	7.24914*E* − 05	*C1qc; Fcgr3a; Tnf; C1s; Cd86; Fcgr1a; Fcgr2b; RT1-DMa; C6; Cd40; LOC498276; C1qb *
4670	Leukocyte transendothelial migration	7.3107*E* − 05	*Ncf4; Mmp9; Cldn14; Msn; Cldn23; Esam; RGD1309537; Rac2; Itgam; Itgb2; Icam1 *
4650	Natural killer cell mediated cytotoxicity	0.000108813	*Fcgr3a; Shc1; Tyrobp; Tnf; Lcp2; Ptpn6; Rac2; Itgb2; Icam1; Fcer1g *
5320	Autoimmune thyroid disease	0.000149638	*RT1-EC2; RT1-A2; RT1-A1; Cd86; RT1-CE5; RT1-DMa; Cd40; RT1-CE2 *
4672	Intestinal immune network for IgA production	0.000155052	*Il6; Cd86; Tgfb1; RT1-DMa; Ltbr; Cd40 *
5310	Asthma	0.000428565	*Tnf; RT1-DMa; Cd40; Fcer1g *
5020	Parkinson's disease	0.000474519	*C1qc; Il6; Il1b; C6; C1qb *
4510	Focal adhesion	0.000611017	*Jun; Flna; Shc1; Tnc; Birc3; Col4a1; Flnc; Itga5; RGD1309537; Rac2; Col4a2; Thbs4; Spp1 *
4630	Jak-STAT signaling pathway	0.000690892	*Myc; Il6; Ptpn6; Clcf1; Il4ra; Stat3; Il13ra1; Socs3; Il11; Csf2rb; Jak3 *
4666	Fc gamma R-mediated phagocytosis	0.001026538	*Ptprc; Hck; Fcgr1a; Arpc1b; Sphk1; Rac2; Fcgr2b; LOC498276 *
4115	p53 signaling pathway	0.001031142	*Gadd45g; Serpine1; Igfbp3; Gadd45b; RGD1566319; Gadd45a; LOC298795 *
5200	Pathways in cancer	0.001310312	*Ret; Myc; Il6; Jun; Mmp9; Runx1; Spi1; Birc3; Col4a1; Fgf2; Rac2; Fos; Col4a2; Ptgs2; Nfkb2; Tgfb1; Stat3; Nfkbia *
5410	Hypertrophic cardiomyopathy (HCM)	0.001575221	*Il6; Tnf; Tpm4; Itga5; Des; Prkab2; Tgfb1 *
5340	Primary immunodeficiency	0.002241893	*Ptprc; Tap1; Cd40; Jak3 *
5221	Acute myeloid leukemia	0.003919315	*Myc; Runx1; Eif4ebp1; Spi1; Stat3 *
4210	Apoptosis	0.004080222	*Il1b; Tnf; Birc3; Nfkbia; Tnfrsf1a; Myd88; Csf2rb *
4662	B cell receptor signaling pathway	0.004536991	*Jun; Ptpn6; Rac2; Fos; Fcgr2b; Nfkbia *
4110	Cell cycle	0.007661902	*Gadd45g; Myc; Mcm3; Gadd45b; Tgfb1; Gadd45a; LOC298795; Mcm6 *
4622	RIG-I-like receptor signaling pathway	0.008260836	*Rnf125; Tnf; Cxcl10; Trim25; Nfkbia *
5222	Small cell lung cancer	0.008411479	*Myc; Birc3; Col4a1; Col4a2; Ptgs2; Nfkbia *
5160	Hepatitis C	0.00890146	*Irf1; Cldn14; Tnf; Cldn23; Stat3; Nfkbia; Socs3; Tnfrsf1a *
4660	T cell receptor signaling pathway	0.010477739	*Ptprc; Jun; Tnf; Lcp2; Ptpn6; Fos; Nfkbia *
5414	Dilated cardiomyopathy	0.010752535	*Tnf; Tpm4; Adcy4; Itga5; Des; Tgfb1 *
5210	Colorectal cancer	0.011794184	*Myc; Jun; Rac2; Fos; Tgfb1 *
5220	Chronic myeloid leukemia	0.015301029	*Myc; Shc1; Runx1; Tgfb1; Nfkbia *
4370	VEGF signaling pathway	0.017300772	*Nos3; Hspb1; Sphk1; Rac2; Ptgs2 *
4144	Endocytosis	0.025647683	*RT1-EC2; Ret; Ehd2; RT1-A2; RT1-A1; Hspa2; Dab2; Tgfb1; Hspa1b; RT1-CE5; RT1-CE2 *
4350	TGF-beta signaling pathway	0.030013655	*Myc; Tnf; Bmp7; Tgfb1; Thbs4 *
4012	ErbB signaling pathway	0.030013655	*Myc; Jun; Shc1; Eif4ebp1; Hbegf *
4141	Protein processing in endoplasmic reticulum	0.038063498	*Hspa2; Dnaja1; Hspa1b; Cryaa; Cryab; Hsph1; Sec24a; Ppp1r15a *
4930	Type II diabetes mellitus	0.045976221	*Tnf; Hk2; Socs3 *
5100	Bacterial invasion of epithelial cells	0.046356305	*Shc1; Arpc1b; Itga5; Hcls1 *

**Table 4:** The top 10 downregulated genes of the model group versus the sham group.

Gene symbol	Gene description	log_2_⁡(FC)	*P* value	*P*.adjust
*Gpr88 *	G-protein coupled receptor 88	−2.24907	0.00163	0.02984
*Rgs9 *	Regulator of G-protein signaling 9	−2.17074	0.00035	0.01507
*Enthd1 *	ENTH domain containing 1	−1.95721	0.00005	0.00606
*Olr59 *	Olfactory receptor 59	−1.90729	0.00238	0.03558
*P2ry12 *	Purinergic receptor P2Y, G-protein coupled, 12	−1.85441	0.00116	0.02520
*Degs2 *	Delta(4)-desaturase, sphingolipid 2	−1.85094	0.00198	0.03247
*Pde10a *	Phosphodiesterase 10A	−1.78373	0.00000	0.00012
*Neu2 *	Sialidase 2 (cytosolic sialidase)	−1.78245	0.00170	0.03040
*Adora2a *	Adenosine A2a receptor	−1.76237	0.00003	0.00478
*Slc22a6 *	Solute carrier family 22 (organic anion transporter), member 6	−1.74866	0.00002	0.00416

**Table 5:** The enriched pathways of the downregulated genes of the model group versus the sham group.

KEGG ID	Description	*P* value	Gene name
4260	Cardiac muscle contraction	1.19216*E* − 07	*Tnnt2; Myh7; Myh6; Atp1a1; Atp1a2; Tnni3; Cox8b; Cacng3 *
4020	Calcium signaling pathway	2.71115*E* − 07	*Plcb1; Cckbr; Drd1a; Tacr3; Slc8a2; Camk4; Adra1d; Atp2b2; Itpr1; Pde1b; Adora2a *
4971	Gastric acid secretion	8.46439*E* − 06	*Plcb1; Cckbr; Kcnk2; Atp1a1; Atp1a2; Itpr1 *
4970	Salivary secretion	1.20354*E* − 05	*Plcb1; Adra1d; Atp2b2; Atp1a1; Atp1a2; Itpr1 *
5410	Hypertrophic cardiomyopathy (HCM)	1.42423*E* − 05	*Tnnt2; Itgb6; Myh7; Myh6; Tnni3; Cacng3 *
4080	Neuroactive ligand-receptor interaction	2.28164*E* − 05	*Cckbr; Drd2; Drd1a; Grm3; Tacr3; Adra1d; Chrm4; Gria3; Htr1d; Adora2a; Adora3 *
5414	Dilated cardiomyopathy	2.66995*E* − 05	*Tnnt2; Itgb6; Myh7; Myh6; Tnni3; Cacng3 *
4360	Axon guidance	0.000275226	*Cxcl12; Ephb3; Robo2; Ngef; Lrrc4c; Epha4 *
4972	Pancreatic secretion	0.000605555	*Plcb1; Atp2b2; Atp1a1; Atp1a2; Itpr1 *
600	Sphingolipid metabolism	0.000811033	*Smpd3; Degs2; Neu2 *
4976	Bile secretion	0.000867651	*Slc22a8; Slco1a2; Atp1a1; Atp1a2 *
4974	Protein digestion and absorption	0.001102164	*Slc8a2; Col11a2; Atp1a1; Atp1a2 *
4270	Vascular smooth muscle contraction	0.001284378	*Plcb1; Ramp1; Adra1d; Itpr1; Adora2a *
4540	Gap junction	0.001989051	*Plcb1; Drd2; Drd1a; Itpr1 *
4720	Long-term potentiation	0.005104314	*Plcb1; Camk4; Itpr1 *
4730	Long-term depression	0.005650817	*Plcb1; Itpr1; Gria3 *
4070	Phosphatidylinositol signaling system	0.007185071	*Plcb1; Dgkb; Itpr1 *
4062	Chemokine signaling pathway	0.009027556	*Cxcl12; Plcb1; Rasgrp2; Gng7; Cx3cl1 *
4530	Tight junction	0.045528155	*Cldn10; Myh7; Myh6 *

**Table 6:** The upregulated genes of the acupuncture group versus the control group.

Gene symbol	Gene description	log_2_⁡(FC)	*P* value	*P*.adjust
*Tph1 *	Tryptophan hydroxylase 1	3.71189	0.04627	0.43944
*Loc684158 *	Similar to chromosome 1 open reading frame 36	1.44377	0.00892	0.43944
*Ccdc19 *	Coiled-coil domain containing 19	1.01508	0.03612	0.43944

**Table 7:** Pathway annotation of the upregulated genes of the acupuncture group versus the control group.

Gene ID	Gene name	KEGG ID	KEGG pathways
24848	*Tph1 *	380	Tryptophan metabolism
24848	*Tph1 *	1100	Metabolism

**Table 8:** The top 10 downregulated genes of the acupuncture group versus the control group.

Gene symbol	Gene description	log_2_⁡(FC)	*P* value	*P*.adjust
*Slpil3 *	Antileukoproteinase-like 3	−7.31439	0.01533	0.43944
*Cyp2c6v1 *	Cytochrome P450, family 2, subfamily C, polypeptide 6, variant 1	−7.22349	0.02509	0.43944
*Dmrt2 *	Doublesex and mab-3 related transcription factor 2	−7.00699	0.03210	0.43944
*Rgd1565502 *	*Rgd1565502 *	−7.00010	0.02472	0.43944
*Olr1376 *	Olfactory receptor 1376	−6.87525	0.02725	0.43944
*Loc680428 *	Hypothetical protein *Loc680428 *	−6.84698	0.02102	0.43944
*Rgd1559709 *	Similar to F-box protein 47	−6.59986	0.03060	0.43944
*Olr883 *	Olfactory receptor 883	−6.50528	0.03626	0.43944
*Loc683282 *	Similar to developmental pluripotency-associated 2	−6.47640	0.03546	0.43944
*Olr469 *	Olfactory receptor 469	−6.44318	0.02394	0.43944

**Table 9:** The enriched pathways of the downregulated genes of the acupuncture group versus the control group.

KEGG ID	Description	*P* value	Gene name
4740	Olfactory transduction	0	*Olr851; Olr1029; Olr883; Olr1507; Olr377; Olr1641; Olr259; Olr1652; Olr722; Olr690; Olr1455; Olr1670; Olr807; Olr1616; Olr419; Olr1384; Olr1240; Olr1204; Olr424; Olr415; Olr404; Olr1535; Olr606; Olr1146; Olr1643; Olr447; Olr469; Olr502; Olr1394; Olr1529; Olr1530; Olr135; Olr344; Olr731; Olr639; Olr1314; Olr1311; Olr610; Olr607; Olr1326; Olr575; Olr1365; Olr1376; Olr1549; Olr1588; Olr1600; Olr20; Olr85; Olr80; Olr47; Olr219; Olr171; Olr170; Olr180; Olr121; Olr886; Olr1482; Olr1749; Olr1714; Olr533; Olr695; Olr737; Olr1060; Olr1389; Olr750; Olr758; Olr792 *
