# Supplementary material for: A Microarray Study of Middle Cerebral Occlusion Rat Brain with Acupuncture Intervention
Source: Evid Based Complement Alternat Med. 2015 Mar 10;2015:496932. doi: 10.1155/2015/496932 (PMC4377484; doi:10.1155/2015/496932)
Supplement: Supplementary file 1 — Supplementary figure 1 shows the different expression genes of the model group versus the sham group. Supplementary figure 2 presents the enriched pathways of the upregulated genes of the model group versus the sham group. Supplementary figure 3 presents the enriched pathways of the downregulated genes of the model group versus the sham group. Supplementary figure 4 shows the different expression genes of the acupuncture group versus the control group. Supplementary figure 5 presents the enriched pathways of the downregulated genes of the acupuncture group versus the control group. Supplementary figure 6 gives the melting curves of Tph1 and Olr883 in RT-PCR experiment: (a) Melting curve of Tph1. (b) Melting curve of Olr883. [file 496932.f1.pdf]

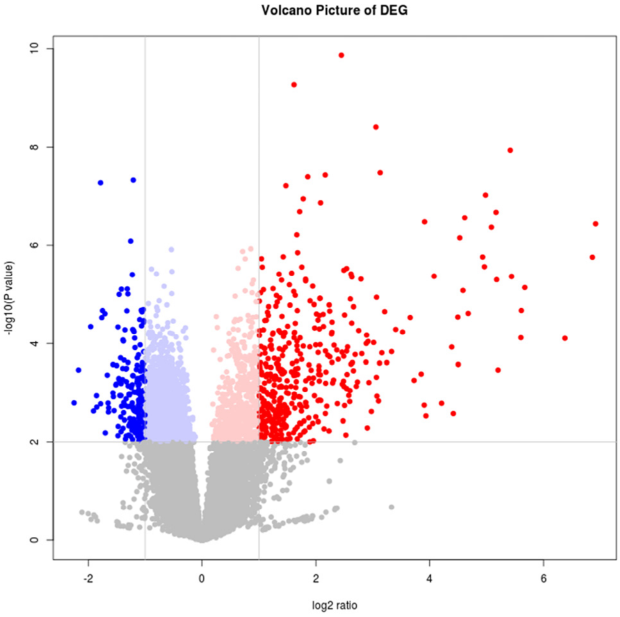

Figure 1: Different expression genes of the model group vs. the sham group

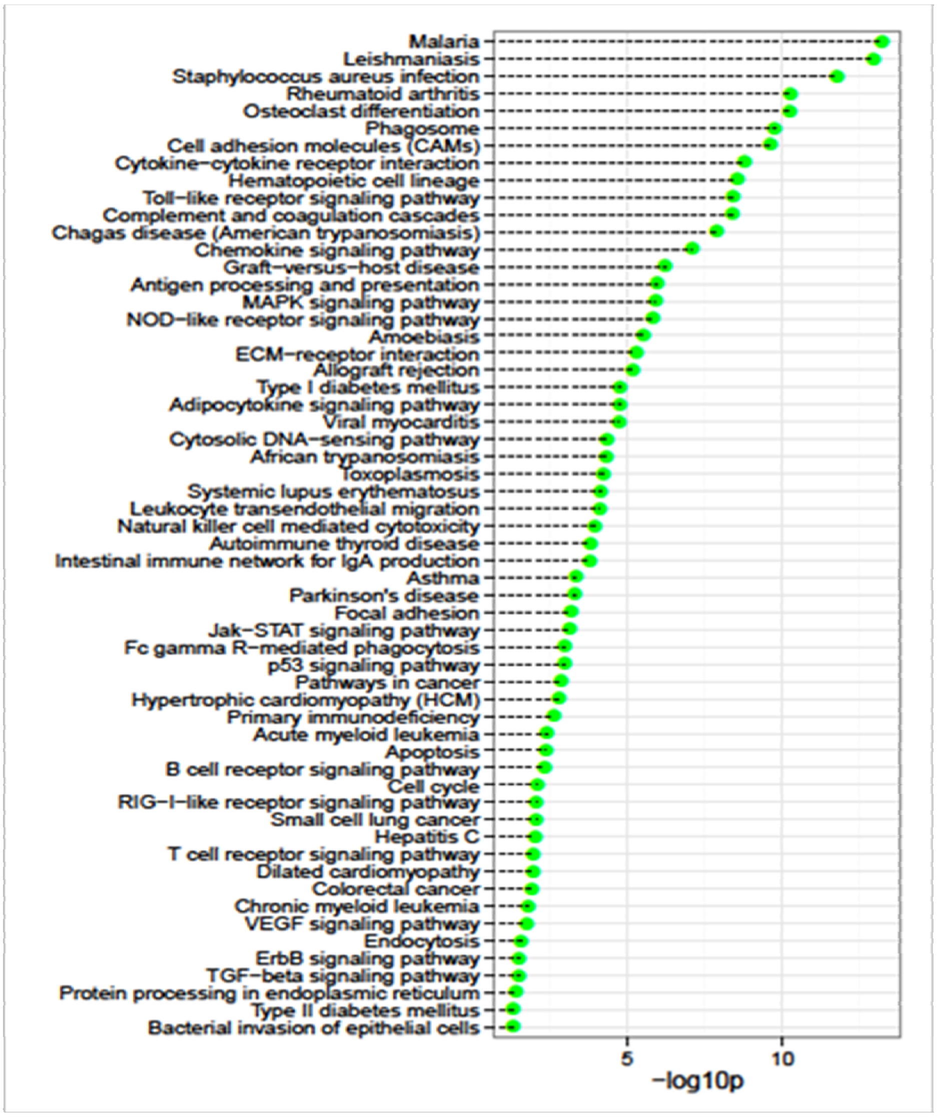

Figure 2: The enriched pathways of the up-regulated genes of the model group vs. the sham group

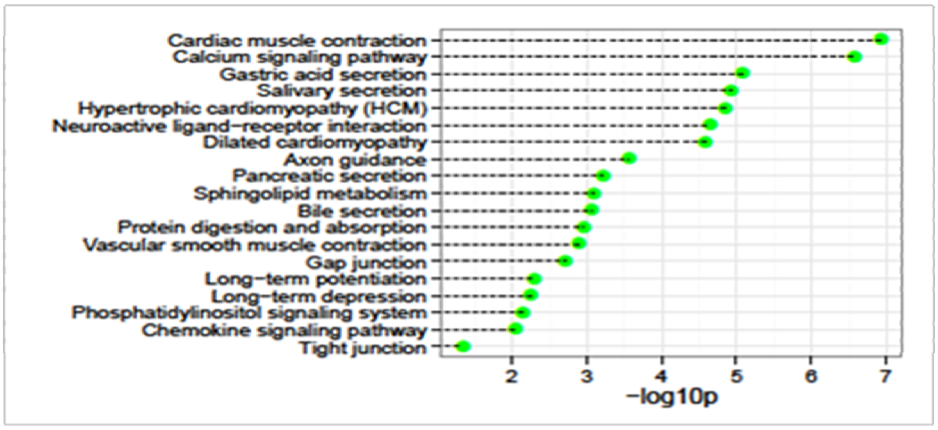

Figure 3: The enriched pathways of the down-regulated genes of the model group vs. the sham group

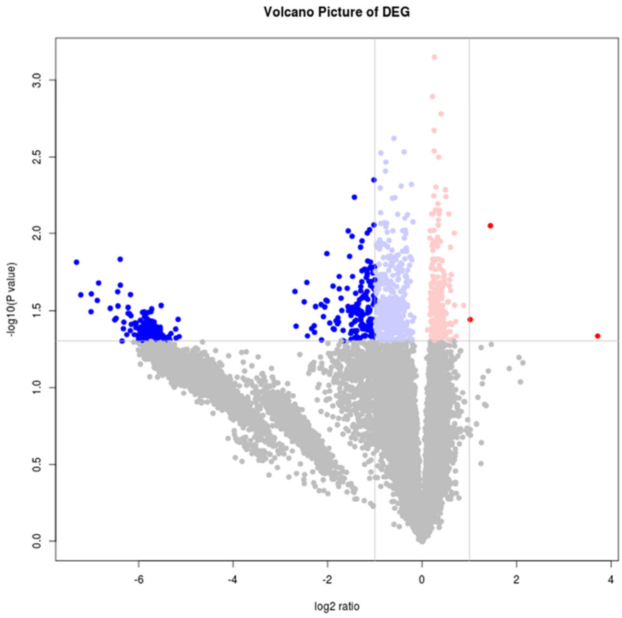

Figure 4: Different expression genes of the acupuncture group vs. the control group

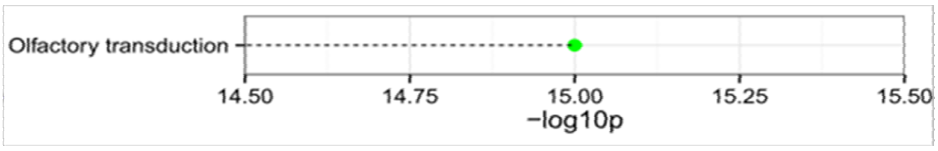

Figure 5: The enriched pathways of the down-regulated genes of the acupuncture group vs. the control group

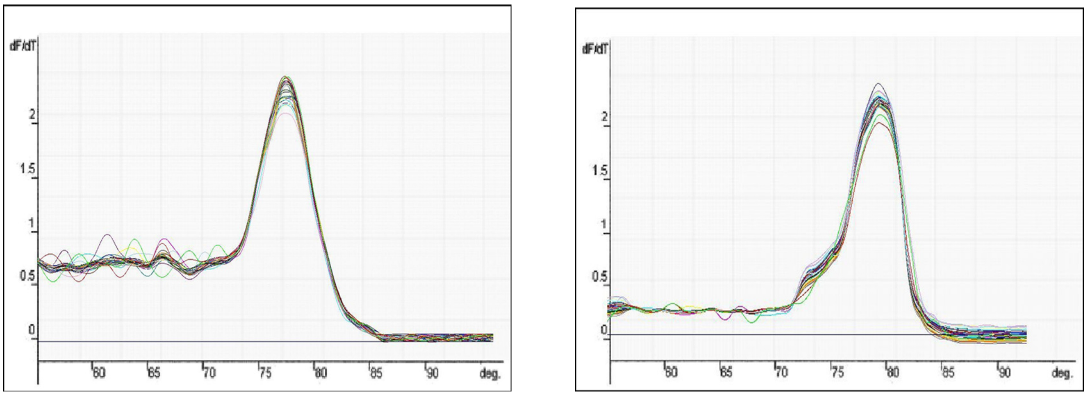

(a) (b)

Figure 6: Analysis of mRNA expression of *Tph1* and *Olr883* by RT-PCR: (a) Melting curve of *Tph1*. (b) Melting curve of *Olr883*.
